# Global Gene Knockout of *Kcnip3* Enhances Pain Sensitivity and Exacerbates Negative Emotions in Rats

**DOI:** 10.3389/fnmol.2019.00005

**Published:** 2019-01-25

**Authors:** Yu-Peng Guo, Yu-Ru Zhi, Ting-Ting Liu, Yun Wang, Ying Zhang

**Affiliations:** ^1^Department of Neurobiology, School of Basic Medical Sciences and Neuroscience Research Institute, Key Laboratory for Neuroscience, Ministry of Education and Ministry of National Health, Peking University, Beijing, China; ^2^PKU-IDG/McGovern Institute for Brain Research, Peking University, Beijing, China

**Keywords:** KChIP3, nociceptive pain, inflammatory pain, conditioned place aversion, anxiety, depression, negative emotions, RNA-Seq analysis

## Abstract

The Ca^2+^-binding protein Kv channel interacting protein 3 (KChIP3) or downstream regulatory element antagonist modulator (DREAM), a member of the neuronal calcium sensor (NCS) family, shows remarkable multifunctional properties. It acts as a transcriptional repressor in the nucleus and a modulator of ion channels or receptors, such as Kv4, NMDA receptors and TRPV1 channels on the cytomembrane. Previous studies of *Kcnip3*^-/-^ mice have indicated that KChIP3 facilitates pain hypersensitivity by repressing *Pdyn* expression in the spinal cord. Conversely, studies from transgenic daDREAM (dominant active DREAM) mice indicated that KChIP3 contributes to analgesia by repressing *Bdnf* expression and attenuating the development of central sensitization. To further determine the role of KChIP3 in pain transmission and its possible involvement in emotional processing, we assessed the pain sensitivity and negative emotional behaviors of *Kcnip3*^-/-^ rats. The knockout rats showed higher pain sensitivity compared to the wild-type rats both in the acute nociceptive pain model and in the late phase (i.e., 2, 4 and 6 days post complete Freund’s adjuvant injection) of the chronic inflammatory pain model. Importantly, *Kcnip3*^-/-^ rats displayed stronger aversion to the pain-associated compartment, higher anxiety level and aggravated depression-like behavior. Furthermore, RNA-Seq transcriptional profiling of the forebrain cortex were compared between wild-type and *Kcnip3*^-/-^ rats. Among the 68 upregulated genes, 19 genes (including *Nr4a2, Ret, Cplx3, Rgs9*, and *Itgad*) are associated with neural development or synaptic transmission, particularly dopamine neurotransmission. Among the 79 downregulated genes, 16 genes (including *Col3a1, Itm2a, Pcdhb3, Pcdhb22, Pcdhb20, Ddc*, and *Sncaip*) are associated with neural development or dopaminergic transmission. Transcriptional upregulation of *Nr4a2, Ret, Cplx3* and *Rgs9*, and downregulation of *Col3a1, Itm2a, Pcdhb3* and *Ddc*, were validated by qPCR analysis. In summary, our studies showed that *Kcnip3*^-/-^ rats displayed higher pain sensitivity and stronger negative emotions, suggesting an involvement of KChIP3 in negative emotions and possible role in central nociceptive processing.

## Introduction

Pain is defined as a distressing experience associated with actual or potential tissue damage with sensory, emotional, cognitive, and social components (Williams and Craig, 2016). Clinically, chronic pain induced by various factors, including tissue inflammation, nerve damage, viral infection, and metabolic disorders, causes patients to suffer spontaneous pain, hyperalgesia and allodynia. At the same time, chronic pain induces a strong emotional response, making it aversive and commonly comorbid with anxiety or depressive disorders. Furthermore, a reciprocal facilitatory effect exists between pain sensitivity and negative emotions. However, chronic pain complaints are generally poorly served by existing therapies (Yekkirala et al., 2017). These patients often do not receive adequate and effective treatment due to limited efficacy or dose-limiting side effects of the current analgesics. Therefore, an in-depth understanding of the molecular mechanisms underlying the development of chronic pain is of significance for the development of innovative analgesics.

Downstream regulator element antagonist modulator (DREAM), a member of the neuronal calcium sensor (NCS) family that contains four Ca^2+^-binding EF-hand motifs, was shown to be a critical transcriptional repressor for pain modulation (Cheng et al., 2002). It represses gene expression as a tetramer via direct binding to the downstream regulatory element (DRE) site containing the central core sequence GTCA (Ledo et al., 2000). Increased intracellular Ca^2+^ concentration can prevent binding of DREAM to the DRE site and derepress DRE-dependent gene expression. Functional expression of DREAM in cortex, hippocampus, cerebellum, spinal cord, dorsal root ganglion (DRG), pineal gland, thyroid and blood cells was validated, and numerous target genes of DREAM were identified, including *Fos, Pdyn* (prodynorphin), *Slc8a3* (solute carrier family 8 member A3), *Npas4* (neuronal PAS domain protein 4) and *Bdnf* (brain-derived neurotrophic factor) (Table 1) (Carrion et al., 1998, 1999; Sanz et al., 2001, 2002; Link et al., 2004; Rivas et al., 2004; D’Andrea et al., 2005; Gomez-Villafuertes et al., 2005; Savignac et al., 2005; Venn et al., 2008; Dierssen et al., 2012; Mellstrom et al., 2014; Benedet et al., 2017). Actually, DREAM is identical to KChIP3 (Kv4 channel interacting protein 3), a member of the KChIPs family, which is composed of KChIP1, 2, 3 and 4. KChIPs interact with the Kv4 channels and modulate A-type potassium currents in a Ca^2+^-dependent manner (An et al., 2000; Anderson et al., 2010). Our recent work revealed the regulation of NMDA receptors and TRPV1 channels by KChIP3/DREAM (Zhang et al., 2010; Tian et al., 2018). Herein, we will refer to the protein KChIP3 for consistency with its gene name *Kcnip3*.

**Table 1:** Target genes modulated by transcriptional repressor KChIP3/DREAM.

Gene name	Tissue/cell type	Reference
*Fos*	Brain	Carrion et al., 1999 and Mellstrom et al., 2014
*Pdyn*	Spinal cord and brain	Carrion et al., 1998, 1999
*Hrk*	Hematopoietic progenitor cells	Sanz et al., 2001, 2002
*Aanat, Crem, Fosl2*	Pineal gland	Link et al., 2004
*Tg, Pax8, Foxe1*	Thyroid	Rivas et al., 2004 and D’Andrea et al., 2005
*Il2, Il4, Ifng*	T-lymphocyte	Savignac et al., 2005
*Slc8a3*	Cerebellar granules	Gomez-Villafuertes et al., 2005 and Venn et al., 2008
*Mid1*	Cerebellum	Dierssen et al., 2012
*Npas4, Nr4a1, Mef2C, Per3, JunB, Sox11, Egr2, Mbd4, Bdnf, Kcnip3*	Hippocampus	Mellstrom et al., 2014
*Mgll, Ctsl*	Trigeminal ganglia	Benedet et al., 2017

The role of KChIP3 in pain modulation was studied by both genetic deletion and transgene-mediated overexpression methods. *Kcnip3*^-/-^ mice displayed markedly reduced responses in models of acute thermal, mechanical, and visceral pain and in models of chronic neuropathic and inflammatory pain (Cheng et al., 2002). Elevated levels of *Pdyn* mRNA and dynorphin A peptide in the spinal cord contributed to the reduction of pain responses. Subsequently, transgenic mice expressing a constitutively active DREAM (daDREAM) mutant displayed a biphasic pain response. The thermal pain reaction was enhanced under basal conditions, while hypoalgesia was observed under inflammatory pain conditions (Rivera-Arconada et al., 2010). Sustained repression of the *Bdnf* gene impaired the development of central sensitization during inflammatory pain. In addition, recent studies from our group indicated that *Kcnip3*^-/-^ rats showed aggravated thermal hyperalgesia in the complete Freund’s adjuvant (CFA)-induced inflammatory pain model (Tian et al., 2018). Taken together, these findings demonstrated the complex regulatory roles of KChIP3 in nociceptive processing.

In the current studies, we performed a series of behavioral tests to investigate the changes in acute and chronic pain responses and negative emotions caused by *Kcnip3* gene deletion. Our results showed that *Kcnip3*^-/-^ rats displayed an enhanced response to acute and chronic pain stimuli and stronger pain-induced aversion and negative emotions. Finally, the potential novel target genes of KChIP3 were revealed by RNA-Seq analysis and validated by quantitative real-time polymerase chain reaction (qPCR).

## Materials and Methods

### Antibodies

For Western blot, rabbit anti-KChIP3 (N-terminal 1–20 amino acids) polyclonal antibody was custom-made by GL Biochem Ltd. (Shanghai, China). Mouse anti-pan KChIP (1/2/4) monoclonal antibody (clone K55/82) was purchased from Millipore (Temecula, CA, United States). Mouse anti-β-actin monoclonal antibody was purchased from Zhongshan Jinqiao Biotechnology Ltd. (Beijing, China). Horseradish peroxidase (HRP)-conjugated secondary antibodies, including goat anti-rabbit IgG and goat anti-mouse IgG, were purchased from Santa Cruz Biotechnology (Dallas, TX, United States).

### Animals

**FIGURE 1:**
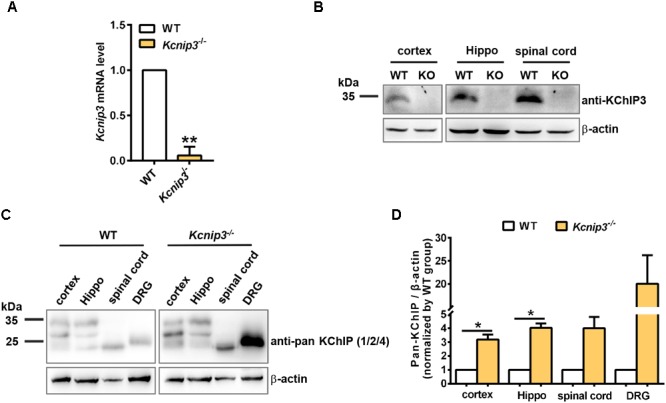
Validation of *Kcnip3* gene knockout in *Kcnip3*^-/-^ rats by quantitative real-time PCR (qPCR) and Western blot. **(A)** qPCR analysis of *Kcnip3* gene expression in the cerebral cortex of wild-type (WT) and *Kcnip3*^-/-^ rats. ^∗∗^*p* < 0.01, paired *t*-test. **(B)** Western blot analysis of KChIP3 protein expression in the central nervous system of wild-type and *Kcnip3*^-/-^ rats. KO, knockout; Hippo, hippocampus. **(C)** Western blot analysis of KChIP1, 2 and 4 expression in the peripheral and central nervous system of wild-type and *Kcnip3*^-/-^ rats under normal condition. DRG, dorsal root ganglion. **(D)** Relative quantification of KChIP1, 2 and 4 protein levels in the peripheral and central nervous system of wild-type and *Kcnip3*^-/-^ rats. *n* = 3 for both groups. ^∗^*p* < 0.05, paired *t*-test.

Male Sprague–Dawley rats weighing 250–280 g at the start of the experiments were used. *Kcnip3*^-/-^ rats were generated by the Nanjing Biomedical Research Institute of Nanjing University (Nanjing, China). Deletion of exon 2 of the *Kcnip3* gene using the CRISPR/Cas9 system induces a frameshift mutation. Null *Kcnip3* gene expression in the knockout rats was verified by qPCR analysis (Figure 1A) and Western blot (Figure 1B). Targeted deletion of *Kcnip3* gene and possible off-target effects of CRISPR/Cas9 were examined by PCR analysis (Supplementary Figure S1). Animals were housed under controlled conditions (22 ± 2°C temperature, 55 ± 5% humidity and a 12:12 light/dark cycle) and had free access to food and water.

The animals were handled 3 days before all the experiments. The experiments were carried out in accordance with the recommendations of the Guidelines of the International Association for the Study of Pain. The protocol was approved by the Animal Care and Use Committee of Peking University (permit number: LA2015074). The behavioral tests were performed in a double-blinded manner by two experimenters. One experimenter was responsible for grouping and numbering the rats. The other one who took charge of the behavioral tests was unaware of the genotypes in the whole experiment.

### qPCR

Total RNA was extracted from the forebrain cortex and purified using the EASYspin Kit (Aidlab, Beijing, China). RNA concentration and purity were measured using a NanoDrop 2000c spectrophotometer (Thermo Scientific, Waltham, MA, United States). SuperScript III reverse transcriptase (Invitrogen, Carlsbad, CA, United States) was used for reverse transcription of RNA into cDNA according to the manufacturer’s instructions.

Quantitative PCR was performed using the ABI 7500 instrument (Applied Biosystems, Foster City, CA, United States). SYBR Green 2× PCR Master Mix (Toyobo, Osaka, Japan) was used for the PCR reaction. The primers are listed in Supplementary Table S3. The reaction conditions were set as follows: incubation at 95°C for 1 min, 40 cycles of 95°C for 15 s, 60°C for 15 s, 72°C for 30 s. Lastly, melting curve analysis was performed with 95°C for 15 s, 60°C for 1 min and 95°C for 15 s. Ct values were defined as the number of PCR cycles at which the fluorescence signals were detected. The relative expression levels of *Kcnip3* were calculated using the 2^-ΔΔCt^ method and were normalized by *Gapdh*. Each sample was measured in triplicate.

### Western Blot Analysis

Naïve wild-type rats or *Kcnip3*^-/-^ rats were deeply anesthetized with 1% sodium pentobarbital. The cortex, hippocampus, spinal cord and bilateral L4–L5 DRG were quickly removed and immediately homogenized in ice-cold lysis buffer (Tiangen Biotech, Beijing, China). The homogenates were centrifuged at 12,000 × *g* for 5 min at 4°C and the supernatants were analyzed. Protein concentrations were measured using a BCA assay kit (Thermo Scientific). Next, 50 μg of each sample was boiled for 5 min with SDS-PAGE sample buffer, subjected to SDS-PAGE using 12% running gels, and transferred onto nitrocellulose membranes. The membranes were blocked with 5% non-fat milk in TBST (50 mM Tris-HCl, pH 7.5, 150 mM NaCl, and 0.05% Tween 20) for 1 h at room temperature and then incubated overnight at 4°C with the appropriate primary antibody [anti-KChIP3, 1:100; anti-pan KChIP(1/2/4) antibody, 1:500; β-actin, 1:1,000]. The blots were then washed with TBST three times for 10 min each time. Next, the membranes were incubated with the appropriate horseradish peroxidase-conjugated secondary antibody for 1 h at room temperature. Finally, the blots were developed with a lightening chemiluminescence kit (Santa Cruz Biotechnology).

### RNA-Sequencing and Data Analysis

RNA isolation was performed as described above. Genomic DNA was removed by gDNA removal column provided in the kit. An Agilent RNA Nano Kit and an Agilent 2100 Bioanalyzer (Santa Clara, CA, United States) were used for RNA integrity and concentration detection. For each sample, 5 μg of total RNA was used to construct the Illumina sequencing libraries according to the manufacturer’s instructions. The libraries were sequenced using the Illumina HiSeq X Ten platform to generate high-quality paired-end reads of 150 nt.

*Rattus norvegicus* genome sequences and annotated gene models were downloaded from ENSEMBL (Rnor6). Raw sequencing reads were first processed to remove adaptors and low-quality bases using Fastqc and Trimmomatic (Bolger et al., 2014) and then aligned to reference genome sequences using STAR (2.5.2b) with gene annotation indexed (Dobin and Gingeras, 2015). The mapping quality and saturation analysis were performed using RSeQC (Wang et al., 2016). Differentially expressed genes were identified using DESeq2 (Love et al., 2014) with absolute log_2_ transformed fold change (FC) value >0.58 and multiple-testing adjusted *p*-value (also known as false discovery rate, FDR) <0.05. Comparison analysis and plots were performed using in-house transcripts and online plot tools^1^ based on Python and R.

The sequencing data have been uploaded in the website of BIG Data Center in Beijing Institute of Genomics of Chinese Academy of Sciences (Beijing, China). The assigned accession number of the submission is CRA001181.

### CFA-Induced Inflammatory Pain Model

Paw inflammation was induced by intraplantar injection of 100 μl CFA (Sigma-Aldrich, St. Louis, MO, United States) into the left hindpaw.

### Formalin Test

Before testing, the rats were allowed to acclimatize to the experimental environment for 30 min. Then, the planta of left hindpaw received subcutaneous injection of 100 μl 5% formalin, which was prepared by dilution with 0.9% saline. Then the number of flinches and the time spent licking the injected paw were recorded for 60 min by a digital camera.

### Hot Plate Test

The rats were placed on a hot plate (Bioseb, United States) to adapt to the environment for 15 min before testing. During the test, the temperature of the hot plate was stabilized at 52°C. The latency to lick the hindpaw or jumping behavior was recorded by a digital camera. To avoid tissue injury, a cut-off time was set at 30 s.

### Cold Plate Test

Adaptation to the environment is performed according to the process mentioned above. During the test, the temperature of the cold plate (Bioseb, United States) was stabilized at 4°C. The number of paw elevations within 1 min was recorded by a digital camera. To avoid tissue injury, a cut-off time was set at 60 s.

### Elevated Plus Maze Test

The elevated plus maze apparatus consisted of four arms (50 cm × 10 cm) made of black Plexiglas, two comprising 40-cm-high walls (closed) and two comprising 1.5-cm-high borders (open) and was elevated 73 cm above the floor. Rats were placed at the center of the maze in a room with dim light for 5 min. A video camera fixed above the maze was used to record the movement of each animal. The number of entries and time spent in each arm were scored. The maze was cleaned with absolute ethanol between each rat.

### Open Field Test

The open field apparatus consisted of a clear Plexiglas box (100 cm^3^ × 100 cm^3^ × 40 cm^3^). Each rat was gently placed in the center of the arena and was allowed to explore the area in a room with dim light for 5 min. The cumulative distance traveled and the time spent in the center (60 cm × 60 cm) were recorded using a digital camera above the arena. The arena was cleaned with absolute ethanol between each rat.

### Forced Swimming Test

Rats were gently placed in the open transparent vertical cylindrical container (diameter 20 cm, height 50 cm) containing water (25°C) and allowed to swim for 6 min under normal light. Water depth prevented rats from hitting the bottom of cylinder with their tails or hind limbs. Rat behaviors were videotaped from the side. The immobile time when rats remained floating or motionless with only movements necessary for maintaining balance in the water during the last 4 min of the test was scored. Each rat received a pretest 24 h before the test, during which rats were placed to the cylinder of water for 15 min.

### Sucrose Preference Test

Rats were housed individually and acclimated for 2 days with two bottles of water, followed by two bottles of 2% sucrose for 2 days. Rats were then deprived of water for 24 h and then exposed to a bottle of 2% sucrose and a bottle of water for 2 h in the dark room. Bottle positions were switched after 1 h (for 2 h test). The total consumption of each liquid was recorded, and the sucrose preference was defined as the average sucrose consumption ratio, which was calculated by dividing the total volume of sucrose intake by the total volume of water and sucrose intake.

### Conditioned Place Aversion Test (CPA)

The CPA test was performed in a shuttle box that consisted of two equal-sized compartments with distinct tactile and visual cues (one had four lamplets that formed a square on the wall and a stainless-steel mesh floor, and the other had four lamplets that formed a triangle on the wall and a stainless-steel rod floor) under dim light.

After habituation for 1 day, the experiment was conducted for consecutive 6 days. On day 1 (preconditioning session), rats freely explored the two compartments for 900 s, and the time spent in each compartment was recorded. Rats that spent more than 600 s or that spent more than 80% of the total time (>720 s) in one compartment were eliminated in the following experiments. In later experiments, we chose the compartment in which the rat spent more than 50% of the total time (>450 s) as the pain-paired compartment. On day 2–day 5 (conditioning session), each rat was confined in the non-pain-paired compartment for 1 h following an intraplantar injection of saline (100 μl) into the right hindpaw. Then the rat was given an intraplantar injection of 5% formalin (100 μl) into the left hindpaw and confined in the pain-paired compartment for 1 h. On day 6 (test session), each rat was allowed to explore the two compartments freely, and the time spent in each compartment during the 900-s session was measured. Two compartments were equipped with an overhead camera to track the rat position. The percentage of preference was determined via AnyMaze software.

### Statistical Analysis

All of the data are represented as the mean ± SEM. Comparisons between two groups were performed using Student’s unpaired or paired *t*-test. Comparisons between two groups at different time points were performed using two-way ANOVA with Sidak’s multiple comparisons test. The criterion for statistical significance was *p* < 0.05, and differences were calculated using GraphPad Prism 7.0.

## Results

### Validation of *Kcnip3* Gene Deletion in the *Kcnip3*^-/-^ Rats

*Kcnip3*^-/-^ rats were generated by CRISPR/CAS9-mediated deletion of exon 2 of rat *Kcnip3* gene as described previously (Tian et al., 2018). qPCR experiments using primers spanning exon 1 and exon 2 of the rat *Kcnip3* gene could barely detect the predicted PCR product in the forebrain cortex of knockout rats (Figure 1A), suggesting efficient deletion of the targeted sequence. At the same time, RNA-Seq analysis in the following studies could not detect the presence of transcripts from exon 2 (data not shown). Although transcripts of exon 3–exon 9 did not show difference between the wild-type and knockout rats, their translation is blocked by frameshift mutation in the knockout animal. In addition, Western blot analysis in the cortex, hippocampus and spinal cord tissues indicated the absence of KChIP3 protein, which was detected using the custom-made anti-KChIP3 antibody against KChIP3 N-terminus (Figure 1B). These data validated the efficient targeted deletion of the *Kcnip3* gene.

Compensatory upregulation among family members commonly occurred in the global knockout animal. Therefore, the cortex, hippocampus, spinal cord, and DRG tissues were collected from naïve wild-type or *Kcnip3*^-/-^ rats. Anti-pan KChIP antibody (Pruunsild and Timmusk, 2012), which can recognize KChIP1, 2 and 4, but not KChIP3 (Supplementary Figure S2), was used for the Western blot analysis (Figure 1C). Quantification analysis showed significant upregulation of KChIP1, 2 and 4 protein in the cortex and hippocampal tissues of knockout rats (*p* < 0.05, paired *t*-test). And an increased trend of expression was observed in the spinal cord and DRG tissues. In addition, qPCR analysis was performed to detect the transcripts of *Kcnip1, 2* and *4* (Supplementary Figure S3A). However, no significant difference was observed between the groups. Therefore, compensatory upregulation of KChIP1, 2 and 4 occurs in the post-transcriptional level.

### *Kcnip3*^-/-^ Rats Display Increased Pain Sensitivity in Both Acute Nociceptive and Chronic Pain Models

In general, *Kcnip3*^-/-^ rats appear healthy and normal and no obvious abnormality in their motor activity and behaviors were observed. Although the knockout rats would be slightly smaller in the late phase of growth, no obvious weight difference was observed around 8 weeks, when the behavioral tests were performed.

**FIGURE 2:**
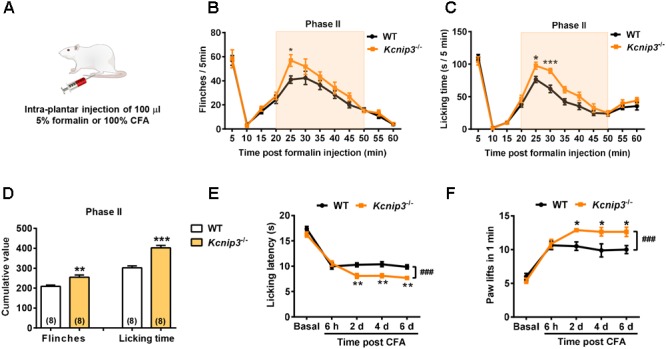
Increased pain sensitivity after intraplantar injection of formalin or complete Freund’s adjuvant (CFA) in *Kcnip3*^-/-^ rats. **(A)** Schematic diagram of the establishment of formalin or CFA-induced pain models. **(B,C)** Flinching counts **(B)** and licking duration **(C)** of hindpaw at 5-min intervals for 1 h in the formalin pain model of wild-type and *Kcnip3*^-/-^ rats. *n* = 8 for both groups. ^∗^*p* < 0.05, ^∗∗∗^*p* < 0.001, two-way repeated-measures ANOVA followed by Sidak’s multiple comparisons test. **(D)** Analysis of the cumulative number of flinching behaviors (left) and licking time (right) of hindpaw in the second phase (20–50 min post injection) of formalin pain model of wild-type and *Kcnip3*^-/-^ rats. ^∗∗^*p* < 0.01, ^∗∗∗^*p* < 0.001, unpaired *t*-test. **(E)** Time course of hindpaw licking latency in the 52°C hot plate test in the CFA pain model of wild-type and *Kcnip3*^-/-^ rats. *n* = 8 for both groups. ^∗∗^*p* < 0.01, comparison between the genotypes at the indicated time points; ^###^*p* < 0.001, comparison between the two curves, two-way repeated-measures ANOVA followed by Sidak’s multiple comparisons test. **(F)** Time course of lifting counts of hindpaw in the 4°C cold plate test within 1 min post CFA injection in wild-type and *Kcnip3*^-/-^ rats. *n* = 8 for both groups. ^∗^*p* < 0.05, comparison between the genotypes at the indicated time points; ^###^*p* < 0.001, comparison between the two curves, two-way repeated-measures ANOVA followed by Sidak’s multiple comparisons test.

To observe the influence of *Kcnip3* gene knockout on the pain responses of rats, we established acute nociceptive and chronic pain models by intraplantar injection of formalin and CFA, respectively (Figure 2A). The formalin test is a reliable and widely used model of continuous pain with a biphasic response consisting of the first transient phase lasting for the first 10 min and the second phase from 10 to 60 min. The first phase is thought to result from direct activation of primary sensory neurons, whereas the second phase has been proposed to reflect the combined effects of afferent input and central sensitization occurring in the dorsal horn (McNamara et al., 2007). We found that both wild-type and *Kcnip3*^-/-^ rats produced a typical biphasic pain response after formalin injection, whereas *Kcnip3*^-/-^ rats exhibited increased flinching behavior (time = 25 min, wild-type: 41.00 ± 3.11, *Kcnip3*^-/-^: 56.88 ± 4.88, *p* < 0.05, two-way ANOVA followed by Sidak’s multiple comparisons test; two groups, *p* < 0.05, two-way repeated measures ANOVA; Figure 2B) and longer duration to lick (time = 25 min, wild-type: 76.38 ± 4.66, *Kcnip3*^-/-^: 97.63 ± 5.42, *p* < 0.05; time = 30 min, wild-type: 61.88 ± 5.76, *Kcnip3*^-/-^: 89.75 ± 3.79, *p* < 0.001; two groups, *p* < 0.001, two-way ANOVA followed by Sidak’s multiple comparisons test; Figure 2C) compared to the wild-type rats in the second phase of the test. Cumulative analysis of the number of flinches and licking time during 20–50 min showed a stronger pain response in *Kcnip3*^-/-^ rats (flinching, wild-type: 208.8 ± 6.886, *Kcnip3*^-/-^: 255.4 ± 11.24, *p* < 0.01, unpaired *t*-test; licking, wild-type: 302.4 ± 9.464, *Kcnip3*^-/-^: 402.4 ± 12.59, *p* < 0.001, unpaired *t*-test; Figure 2D).

We also induced a persistent inflammatory pain model via injection of CFA in the hindpaw and measured the pain responses using the 52°C hot plate and 4°C cold plate tests. There was no significant difference in the basal pain responses between *Kcnip3*^-/-^ rats and the wild-type control. However, after CFA injection the *Kcnip3*^-/-^ rats exhibited significantly decreased licking latency in the hot plate test (time = 2^nd^ day, wild-type: 10.28 ± 0.40, *Kcnip3*^-/-^: 8.09 ± 0.47, *p* < 0.01; time = 4^th^ day, wild-type: 10.39 ± 0.48, *Kcnip3*^-/-^: 8.13 ± 0.40, *p* < 0.01; time = 6^th^ day, wild-type: 9.88 ± 0.40, *Kcnip3*^-/-^: 7.69 ± 0.38, *p* < 0.01; two groups, *p* < 0.001, two-way ANOVA followed by Sidak’s multiple comparisons test; Figure 2E). Similarly, the *Kcnip3*^-/-^ rats showed an increased number of hindpaw lifting within 1 min in the cold plate test (time = 2^nd^ day, wild-type: 10.50 ± 0.63, *Kcnip3*^-/-^: 12.88 ± 0.23, *p* < 0.05; time = 4^th^ day, wild-type: 9.88 ± 0.97, *Kcnip3*^-/-^: 12.63 ± 0.63, *p* < 0.05; time = 6^th^ day, wild-type: 10.00 ± 0.60, *Kcnip3*^-/-^: 12.63 ± 0.68, *p* < 0.05; two groups, *p* < 0.001, two-way ANOVA followed by Sidak’s multiple comparisons test; Figure 2F). Taken together, these data suggested increased pain sensitivity of *Kcnip3*^-/-^ rats in the late phase of inflammatory pain.

### *Kcnip3*^-/-^ Rats Show a Stronger Aversive Response to the Nociceptive Stimuli

**FIGURE 3:**
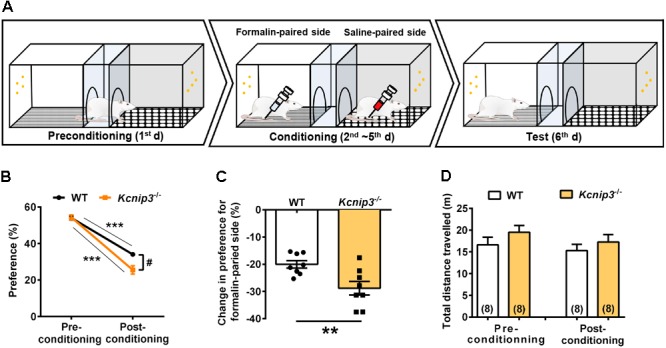
*Kcnip3* gene deletion exacerbates the aversive emotional response of pain. **(A)** Schematic diagram of a three-chamber conditioned place avoidance (CPA) apparatus and training procedure. **(B)** Percentage of the time spent in the formalin-paired compartment before and after the conditioning procedure in wild-type and *Kcnip3*^-/-^ rats. *n* = 8 for both groups. ^∗∗∗^*p* < 0.001, comparison between preconditioning and postconditioning; ^#^*p* < 0.05, comparison between the two curves, two-way repeated-measures ANOVA followed by Sidak’s multiple comparisons test. **(C)** Change in the percentage of the time spent in the formalin-paired compartment after the conditioning procedure in comparison to that prior to the conditioning procedure in wild-type and *Kcnip3*^-/-^ rats. *n* = 8 for both groups. ^∗∗^*p* < 0.01, unpaired *t*-test. **(D)** Total distance traveled before (left, preconditioning) and after (right, postconditioning) the conditioning procedure in wild-type and *Kcnip3*^-/-^ rats. *n* = 8 for both groups. *p* > 0.05, unpaired *t*-test.

Pain consists of sensory-discriminative and negative-affective components, including aversion to pain-associated environments, anxiety and depression. In the following studies, we explored how *Kcnip3* gene knockout affected the emotional responses of pain. First, we performed the CPA test associated with formalin injection (Figure 3A). During the 4-day training session, the rat is confined to one compartment following formalin injection and confined to the opposite compartment following saline injection each day. In the test session, both wild-type and *Kcnip3*^-/-^ rats showed avoidance for the compartment paired with formalin injection, as demonstrated in Figure 3B (wild-type, pre: 54.02 ± 1.21, post: 34.01 ± 1.10, *p* < 0.001; *Kcnip3*^-/-^, pre: 54.31 ± 1.36, post: 25.52 ± 2.30, *p* < 0.001, two-way ANOVA followed by Sidak’s multiple comparisons test). However, *Kcnip3*^-/-^ rats displayed stronger aversion to the formalin-paired compartment (two groups, *p* < 0.05, two-way repeated measures ANOVA; Figure 3B) and exhibited a more profound decrease in preference for the formalin-paired compartment (wild-type: -20.01 ± 1.37%, *Kcnip3*^-/-^: -28.80 ± 2.49%, *p* < 0.01, unpaired *t*-test; Figure 3C). As a control, the amount of locomotor activity between the two groups showed no significant difference before and after the conditioning procedure (Figure 3D). In summary, these data suggest that *Kcnip3* knockout aggravated the aversive response to nociceptive stimuli in rats.

### *Kcnip3*^-/-^ Rats Exhibited Increased Anxiety-Like Behavior Post CFA Injection

**FIGURE 4:**
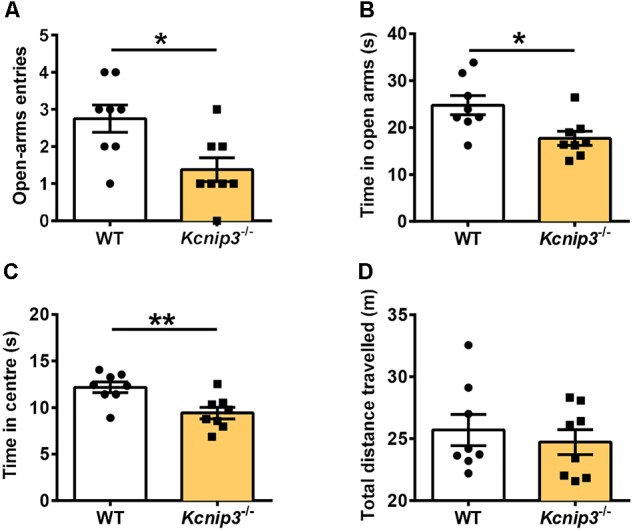
Enhanced anxiety-like behavior in *Kcnip3*^-/-^ rats during inflammatory pain. **(A,B)** The elevated plus-maze test. *Kcnip3*^-/-^ rats made fewer visits to the open arms **(A)** and spent less time in the open arms **(B)** in a 5-min test 1 day after CFA injection compared to the wild-type rats. *n* = 8 for both groups. ^∗^*p* < 0.05, unpaired *t*-test. **(C,D)** The open field test. **(C)** *Kcnip3*^-/-^ rats displayed less time spent in the center of the open field in a 5-min test 1 day after CFA injection compared to the wild-type rats. *n* = 8 for both groups. ^∗∗^*p* < 0.01, unpaired *t*-test. **(D)** Wild-type and *Kcnip3*^-/-^ rats exhibit similar locomotor activity in the open field test 1 day after CFA injection. *n* = 8 for both groups, unpaired *t*-test.

Elevated plus maze and open field tests are routinely used for the assessment of anxiety-related behavior. These tests are based on the naturalistic conflict between the tendency to explore a novel environment and the aversive properties of a brightly lit, open area. Our previous studies indicated that *Kcnip3*^-/-^ rats displayed high basal anxiety levels in the elevated plus maze test (Li et al., 2018). Herein, we examined the anxiety level of *Kcnip3*^-/-^ rats during inflammatory pain. These rats showed decreased open-arm entries (wild-type: 2.75 ± 0.367, *Kcnip3*^-/-^: 1.38 ± 0.32, *p* < 0.05, unpaired *t*-test; Figure 4A) and spent less time in the open arms (wild-type: 24.76 ± 2.04, *Kcnip3*^-/-^: 17.71 ± 1.48, *p* < 0.05, unpaired *t*-test; Figure 4B) in the elevated plus maze test 1 day after CFA injection. In the open field test, *Kcnip3*^-/-^ rats displayed a significant decrease in the time spent in the open center area (wild-type: 12.18 ± 0.58, *Kcnip3*^-/-^: 9.42 ± 0.62, *p* < 0.05, unpaired *t*-test; Figure 4C). In contrast, analysis of the locomotor ability during the open field test showed no difference between the two groups (Figure 4D). Altogether, these results suggest that *Kcnip3* knockout exacerbates inflammatory pain-induced anxiety-like behavior in rats.

### *Kcnip3*^-/-^ Rats Exhibited Increased Depressive-Like Behavior Under Both Normal and Inflammatory Pain Conditions

**FIGURE 5:**
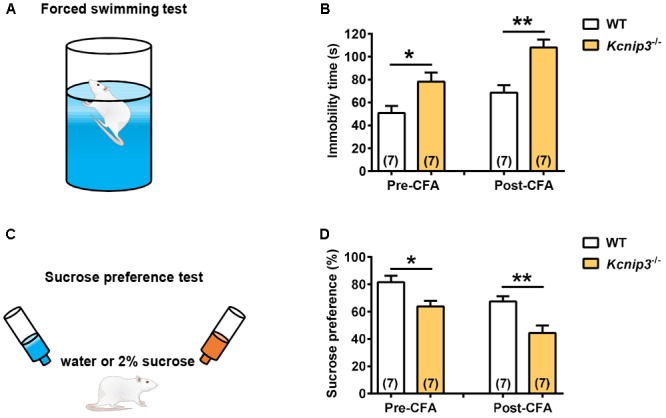
Enhanced depression-like behavior in *Kcnip3*^-/-^ rats under both normal and inflammatory pain conditions. **(A)** Schematic diagram of the forced swimming test. **(B)** Immobility time of wild-type and *Kcnip3*^-/-^ rats before (left) and 1 day after CFA injection (right). *n* = 7 for both groups. ^∗^*p* < 0.05, ^∗∗^*p* < 0.01, unpaired *t*-test. **(C)** Schematic diagram of the sucrose preference test. **(D)** Preference for sucrose, expressed as a percentage of the volume of sucrose intake over the total volume of fluid intake of wild-type and *Kcnip3*^-/-^ rats before (left) and 1 day after CFA injection (right). *n* = 7 for both groups. ^∗^*p* < 0.05, ^∗∗^*p* < 0.01, unpaired *t*-test.

The core symptoms of depression include behavioral despair and anhedonia, a reduced sensitivity to reward. The forced swimming test (Figure 5A) measures the coping strategy of the animal to an acute inescapable stress, and the physical immobility is thought to be an indication of behavioral despair. *Kcnip3*^-/-^ rats showed significantly increased immobility time both under normal conditions (wild-type: 50.86 ± 6.18, *Kcnip3*^-/-^: 78.14 ± 7.98, *p* < 0.05, unpaired *t*-test) and 1 day post CFA injection (wild-type: 68.57 ± 6.48; *Kcnip3*^-/-^: 108.00 ± 7.06, *p* < 0.01, unpaired *t*-test; Figure 5B). In addition, the wild-type showed an increasing trend in the immobility time (pre: 50.86 ± 6.18, post: 68.57 ± 6.48, *p* > 0.05, paired *t*-test) post CFA injection compared to that prior to CFA injection, whereas *Kcnip3*^-/-^ rats showed significantly increased immobility time (pre: 78.14 ± 7.98, post: 108.00 ± 7.06, *p* < 0.05, paired *t*-test), suggesting that *Kcnip3*^-/-^ rats are more vulnerable to depression than wild-type rats.

The sucrose preference test (Figure 5C) represents the anhedonia-like behavioral change. *Kcnip3*^-/-^ rats exhibited lower sucrose preference to 2% solution both under normal conditions (wild-type: 81.57 ± 4.8, *Kcnip3*^-/-^: 63.86 ± 4.11, *p* < 0.05, unpaired *t*-test) and 1 day post CFA injection (wild-type: 67.43 ± 3.88, *Kcnip3*^-/-^: 44.43 ± 5.47, *p* < 0.05, unpaired *t*-test; Figure 5D). At the same time, the wild-type rats showed a decreasing trend in sucrose preference post CFA injection (pre: 81.57 ± 4.83, post: 67.43 ± 3.88, *p* > 0.05, paired *t*-test), whereas the *Kcnip3*^-/-^ rats showed significantly decreased sucrose preference (pre: 63.86 ± 4.11, post: 44.43 ± 5.47, *p* < 0.05, paired *t*-test) post CFA injection compared to that prior to CFA injection, suggesting that *Kcnip3*^-/-^ rats are more susceptible to depression than wild-type rats. Taken together, the above data indicate that *Kcnip3* knockout aggravates anxiety- and depressive-like behaviors in rats. KChIP3 might play an anxiolytic and antidepressant action *in vivo*.

### RNA-Seq Analysis Revealed the Differentially Expressed Genes in *Kcnip3*^-/-^ Rats

**FIGURE 6:**
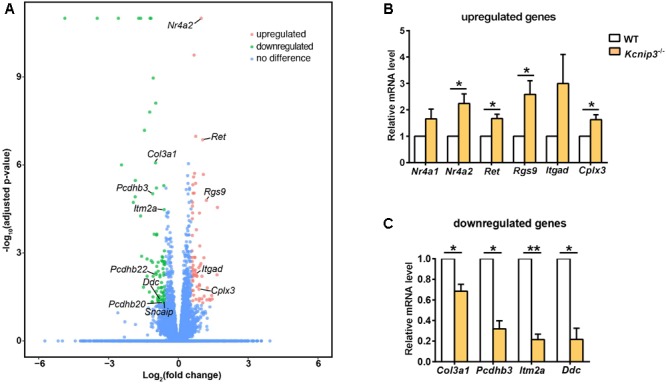
**(A)** Volcano plots illustrating log_10_(adjusted *p*-value) in relation to the log_2_(fold change) for the differentially expressed genes in *Kcnip3*^-/-^ rats compared to wild-type rats. Genes that passed the significance threshold (FDR < 0.05) and the expression cut-off log_2_(fold change) > 0.58 are colored red (upregulated, fold change > 1.5) or green (downregulated, fold change < 0.67), while genes outside this range are colored blue. **(B)** qPCR analysis of upregulated genes in the forebrain cortex of wild-type (WT) and *Kcnip3*^-/-^ rats. *n* = 4–5, ^∗^*p* < 0.05, paired *t*-test. **(C)** qPCR analysis of downregulated genes in the forebrain cortex of WT and *Kcnip3*^-/-^ rats. *n* = 3, ^∗^*p* < 0.05, ^∗∗^*p* < 0.01, paired *t*-test.

To search for differentially expressed genes in *Kcnip3*^-/-^ rats, RNA-Seq transcriptional profiling was performed. The cerebral cortex in the forebrain was collected from four *Kcnip3*^-/-^ rats and four wild-type rats at 8 weeks of age. Using the criteria of FDR < 0.05 and abs(log_2_FC) > 0.58 (upregulated: FC > 1.5; downregulated: FC < 0.67), 68 upregulated genes were identified, and 79 downregulated genes were identified (Figure 6A and Supplementary Tables S1, S2).

Among the upregulated genes, 15 genes are associated with neural development, including *Nr4a2, Ret, Egr3, Rgs9, Bcl11b, Rtn4rl2, Rspo1, Auts2, Scrt2, Itgad, Bag 3, Pcdhgb6, Hmox1, Rfx4* and *Hbegf* (Table 2). Five genes are involved in transcriptional regulation, including *Nr4a2, Egr3, Bcl11b, Scrt2* and *Rfx4*. Genes *Rps27a* and *Sgms2*5 are associated with myelin sheath function. In addition, *Cplx3* is involved in synaptic vesicle exocytosis and neurotransmitter release. Genes *Nr4a2, Rgs9* and *Itgad* are related to dopamine neurotransmission. The *Nr4a2* encoded protein acts as a transcriptional activator and is involved in projection neuron axonogenesis, neuron maturation and migration. In particular, it is associated with dopaminergic neuron differentiation and dopamine biosynthetic processes. *Rgs9* is involved in nervous system development and the dopamine receptor signaling pathway. *Itgad* is related to the negative regulation of dopamine metabolic processes. Consistently, expression of *Crem* and *Fosb*, the known target genes of KChIP3 (Link et al., 2004; Ruiz-DeDiego et al., 2015), were found to be upregulated in *Kcnip3*^-/-^ rats.

**Table 2:** Upregulated genes associated with neural development, neurotransmission or myelin sheath function in the forebrain cortex of *Kcnip3*^-/-^ rat compared to that of wild-type rats.

Gene symbol	Gene name	FDR	log_2_FC	Gene description
*Nr4a2*	Nuclear receptor subfamily 4, group A, member 2	3.91*E*-12	0.98	Transcriptional activator activity
*Ret*	Ret proto-oncogene	1.39*E*-07	1.05	Protein tyrosine kinase activity
*Egr3*	Early growth response 3	1.94*E*-06	0.70	Nucleic acid binding
*Rps27a*	Ribosomal protein S27a	2.57*E*-06	0.66	Structural constituent of ribosome
*Rgs9*	Regulator of G-protein signaling 9	1.61*E*-05	1.20	GTPase activator activity
*Bcl11b*	B-cell CLL/lymphoma 11B	6.82*E*-05	0.60	Transcriptional activator activity
*Rtn4rl2*	Reticulon 4 receptor-like 2	1.43*E*-03	0.72	Protein kinase inhibitor activity
*Rspo1*	R-spondin 1	1.46*E*-03	1.10	Positive regulation of Wnt signaling pathway
*Auts2*	Autism susceptibility 2 protein homolog	3.18*E*-03	0.59	Actin cytoskeleton reorganization; chromatin binding
*Scrt2*	Scratch family transcriptional repressor 2	3.18*E*-03	0.88	Nucleic acid binding
*Itgad*	Integrin subunit alpha D	5.06*E*-03	0.76	Integrin-mediated signaling pathway
*Bag3*	Bcl2-associated athanogene 3	5.47*E*-03	0.67	Cadherin binding
*Pcdhgb6*	Protocadherin gamma subfamily B, 6	5.81*E*-03	0.73	Homophilic cell adhesion via plasma membrane adhesion molecules
*Rfx4*	Regulatory factor X4	6.10*E*-03	0.65	Transcriptional activator activity
*Sgms2*	Sphingomyelin synthase 2	6.23*E*-03	1.01	Sphingomyelin synthase activity
*Hbegf*	Heparin-binding EGF-like growth factor	6.34*E*-03	0.6	Epidermal growth factor receptor signaling pathway
*Hmox1*	Heme oxygenase 1	9.23*E*-03	0.62	Positive regulation of I-kappaB kinase/NF-kappaB signaling
*Cplx3*	Complexin 3	1.73*E*-02	0.89	Synaptic vesicle exocytosis
*Prokr2*	Prokineticin receptor 2	5.00*E*-02	1.05	G-protein coupled receptor activity

Among the downregulated genes, six genes are associated with neural development, including *AABR070317, Col3a1, Itm2a, Aqp1, Pcdhgb8* and *Pcdhgb7* (Table 3). Notably, the cell adhesion molecule genes *Pcdhb3, Pcdhb22* and *Pcdhb20* are associated with chemical synaptic transmission and synapse assembly. The *Ddc* and *Sncaip* genes are related to dopamine and serotonin biosynthetic processes and dopamine metabolic processes, respectively. *Hba-a2* and *Ubc* are associated with myelin sheath function. In addition, the *Nqo2*-encoded protein is involved in the positive regulation of the neuronal apoptotic process and memory deficit.

**Table 3:** Downregulated genes associated with neural development, neurotransmission or myelin sheath function in the forebrain cortex of *Kcnip3*^-/-^ rat compared to that of wild-type rats.

Gene symbol	Gene name	FDR	log_2_FC	Gene description
*AABR07031734.12*	Unknown	1.69*E*-12	–3.49	Homophilic cell adhesion via plasma membrane adhesion molecules
*Col3a1*	Collagen type III alpha 1 chain	8.49*E*-07	–0.98	Extracellular matrix structural constituent
*Pcdhb3*	Protocadherin beta 3	9.59*E*-06	–1.10	Homophilic cell adhesion via plasma membrane adhesion molecules
*Itm2a*	integral membrane protein 2A	3.35*E*-05	–0.61	Negative regulation of amyloid precursor protein biosynthetic process
*Lamc3*	Laminin subunit gamma 3	2.46*E*-04	–0.94	Basement membrane
*Aqp1*	Aquaporin 1	1.30*E*-03	–1.58	Water channel activity
*Hba-a2*	Hemoglobin alpha, adult chain 2	2.08*E*-03	–0.86	Oxygen transport; amyloid-beta binding
*Pcdhgb8*	Protocadherin gamma subfamily B, 8	2.39*E*-03	–0.61	Homophilic cell adhesion via plasma membrane adhesion molecules
*Ubc*	Ubiquitin C	2.51*E*-03	–0.59	Protease binding
*Kcng4*	Potassium voltage-gated channel modifier subfamily G member 4	4.78*E*-03	–0.63	Delayed rectifier potassium channel activity
*Pcdhb22*	Protocadherin beta 22	5.38*E*-03	–0.95	Homophilic cell adhesion via plasma membrane adhesion molecules
*Nqo2*	N-ribosyldihydronicotinamide : quinone reductase 2	6.23*E*-03	–1.08	Dihydronicotinamide riboside quinone reductase activity
*Pcdhgb7*	Protocadherin gamma subfamily B, 7	2.90*E*-02	–0.59	Homophilic cell adhesion via plasma membrane adhesion molecules
*Ddc*	DOPA decarboxylase	3.97*E*-02	–0.71	Aromatic-L-amino-acid decarboxylase activity
*Sncaip*	Synuclein, alpha interacting protein	4.65*E*-02	–0.62	Dopamine metabolic process
*Pcdhb20*	Protocadherin beta 20	4.93*E*-02	–0.67	Homophilic cell adhesion via plasma membrane adhesion molecules

Further, we used qPCR experiments to check the upregulated or downregulated genes caused by *Kcnip3* gene deletion. Gene expression of *Nr4a2, Ret, Rgs9* and *Cplx3* were significantly increased (*Nr4a2*, 2.24 ± 0.37 fold of WT control, *p* < 0.05; *Ret*, 1.67 ± 0.17 fold of WT control, *p* < 0.05; *Rgs9*, 2.59 ± 0.52, *p* < 0.05; *Cplx3*, 1.63 ± 0.19 fold of WT control, *p* < 0.05, paired *t*-test; Figure 6B). The expression of *Nr4a1* and *Itgad* showed an increased trend. Considering the transcriptional repressor activity of KChIP3, these upregulated genes might be the new candidate target genes repressed by KChIP3. However, expression of *Pdyn* and *Bdnf*, the known target genes of KChIP3, did not show upregulation in our analysis (Supplementary Figure S3B). On the other hand, gene expression of *Col3a1, Pcdhb3, Itm2a* and *Ddc* were significantly decreased (*Col3a1*, 0.69 ± 0.07 fold of WT control, *p* < 0.05; *Pcdhb3*, 0.32 ± 0.08 fold of WT control, *p* < 0.05; *Itm2a*, 0.21 ± 0.05, *p* < 0.01; *Ddc*, 0.21 ± 0.11 fold of WT control, *p* < 0.05 or *p* < 0.01, paired *t*-test; Figure 6C). Altogether, results from qPCR analysis validated the upregulated and downregulated genes revealed by RNA-Seq analysis in *Kcnip3*^-/-^ rats.

## Discussion

In the current studies, we performed a series of behavioral tests to observe the changes in pain sensitivity and negative emotions in *Kcnip3*^-/-^ rats. The knockout rats showed increased spontaneous behaviors in the formalin test and enhanced heat hyperalgesia and cold hyperalgesia in the CFA test. Notably, *Kcnip3*^-/-^ rats displayed stronger aversion to the pain-paired compartment in the CPA test and showed higher levels of anxiety and depression post CFA injection. At the same time, the knockout rats are more depressed than the wild-type rats under the basal condition. As negative emotions might aggravate the pain responses, the higher anxiety and depression level in *Kcnip3*^-/-^ rats might contribute to the increased sensitivity to pain. Altogether, our studies provide evidence for the involvement of KChIP3 in negative emotions and possible role in central nociceptive processing.

With respect to the mechanisms of the behavioral changes of *Kcnip3*^-/-^ rats, changes in gene expression associated with neural development and synaptic transmission, particularly dopaminergic neurotransmission, were revealed by RNA-Seq analysis in the forebrain cortex. Further studies are needed to address whether structural changes in the brain during development and abnormalities in dopaminergic neurotransmission occurring in *Kcnip3*^-/-^ rats affect central nociceptive and emotional processing.

### Involvement of KChIP3 in Pain Modulation

The involvement of KChIP3 in pain modulation was first described in *Kcnip3*^-/-^ mice (Cheng et al., 2002). The knockout mice displayed markedly reduced pain behaviors both in models of acute thermal, mechanical, and visceral pain and in models of chronic neuropathic and inflammatory pain. The attenuation of the pain response was ascribed to the elevated level of *Pdyn* expression in the spinal cord. Later, studies in transgenic daDREAM mice (with high expression of the dominant active DREAM in DRG and spinal cord in addition to telencephalon) showed that these mice displayed a biphasic pain response, basal hyperalgesia and reduced hyperalgesic response following peripheral inflammation (Rivera-Arconada et al., 2010). In detail, the daDREAM mice showed an enhanced response to thermal and visceral noxious stimuli in basal conditions. However, they displayed an impaired response to inflammatory pain with milder and shorter-lasting hyperalgesia compared to the wild-type mice. Attenuation of central sensitization due to reduced *Bdnf* gene expression contributed to the hypoalgesic behavior of the transgenic mice. Recently, it was reported that another line of daDREAM mice (with daDREAM expression in trigeminal ganglia) showed a significant increase in the rubbing response in the first and second phases of the formalin test, which might be correlated with decreased expression of *Pdyn* (Benedet et al., 2017). Conversely, the nocifensive response to 4.5% formalin injection in the snoot in *Kcnip3*^-/-^ mice was milder than that in normal mice.

Recent studies from our lab demonstrated that *Kcnip3*^-/-^ rats exhibited aggravated heat hyperalgesia behavior following CFA injection, which was measured by a radiant heat test (Tian et al., 2018). Consistently, current studies using formalin and CFA model of pain showed enhanced pain responses in *Kcnip3*^-/-^ rats. The exact reason for the discrepancy between *Kcnip3*^-/-^ mice and *Kcnip3*^-/-^ rats remains unknown. In addition to the species difference, potential unwanted off-target effects of the CRISPR-Cas9 technology or the compensatory upregulation of KChIP1, 2 and 4 protein following *Kcnip3* gene deletion (Figures 1C,D) might also influence the behavioral phenotype. The region- and time-specific gene deletion method needs to be used to further elucidate the role of KChIP3 in pain transmission.

### Potential Anxiolytic and Antidepressant Effects of KChIP3

Previously, lines of evidence indicated the participation of central KChIP3 in learning and memory in mice. For example, *Kcnip3*^-/-^ mice exhibited remarkably increased short-term memory as well as significantly enhanced long-term memory (Fontan-Lozano et al., 2009). Conversely, contextual fear memory, but not auditory fear memory, was significantly impaired in daDREAM mice (Wu et al., 2010). In addition, the daDREAM mice showed a clear defect in spatial memory and associative learning (Mellstrom et al., 2014). However, the involvement of KChIP3 in emotional processing has attracted less attention.

In fact, previous studies in *Kcnip3*^-/-^ mice demonstrated that they have slightly increased anxiety levels compared to the wild-type controls, and no significant difference was observed compared to the wild-type control (Alexander et al., 2009). Another study in female *Kcnip3*^-/-^ mice also showed that the *Kcnip3* gene deletion did not affect the anxiety level in the ovariectomized mice receiving or not receiving estradiol injections (Tunur et al., 2013). However, both our studies from elevated plus maze and open field tests indicated that *Kcnip3*^-/-^ rats had a higher anxiety level compared to the wild-type rats post CFA injection. Combined with our previous results showing the higher basal anxiety level of *Kcnip3*^-/-^ rats (Li et al., 2018), the potential anxiolytic action of KChIP3 protein was supposed.

In addition to increased anxiety-like behavior, *Kcnip3*^-/-^ rats showed stronger aversion mood in the CPA test and showed more depression-like behavior in the forced swimming test and sucrose preference test both under basal and inflammatory pain conditions. All these data support the relief effect of KChIP3 on negative emotions. However, the target genes or ion channels of regulated by KChIP3 in the above processes need to be investigated in future studies.

### The Emerging Role of KChIP3 in Neural Development

The RNA-Seq analysis in the forebrain cortex revealed that 15 upregulated genes and 6 downregulated genes in *Kcnip3*^-/-^ rats are associated with neural development, implying the potential involvement of KChIP3 in neural development. In detail, *Nr4a, Ret* and *Bcl11b* are related to neuron axonogenesis. *Ret* and *Bcl11b* are associated with neuron differentiation. *Ret, Auts2, Scrt2, Col3a1* and *Aqp1* are involved in neuron migration. *Bcl11b, Rtn4rl2, Bag3* and *Aqp1* contribute to neuron projection. In particular, *Nr4a2, Egr3, Rtn4rl2, Bag3* and *Col3a1* are associated with habenular, peripheral nervous system, corpus callosum, brain and spinal cord, and cerebral cortex development, respectively. *Rfx4* is involved in forebrain, midbrain and dorsal spinal cord development. Altogether, these data support the potential role of KChIP3 in neural development. However, many other genes, such as those related to oxygen transport, iron ion binding and blood cell differentiation, showed changes in their expression after *Kcnip3* deletion. Possible impact of these genes on development could not be excluded.

Previous studies indicated that expression of midline 1 (*Mid1*), a ubiquitin ligase specific for the protein phosphatase 2A, is repressed in daDREAM mice (Dierssen et al., 2012). Related to this, daDREAM mice exhibit a significant shortening of the rostro-caudal axis of the cerebellum and a severe delay in neuromotor development early after birth, suggesting a role of DREAM in cerebellar development. In addition, daDREAM mice showed reduced dendritic basal arborization and spine density in CA1 pyramidal neurons but increased spine density in dendrites in dentate gyrus granule cells in hippocampus (Mellstrom et al., 2016). Recent *in vitro* studies indicated that KChIP3 contributes to neuritogenesis through RhoA inactivation. PC12 cells expressing KChIP3 had increased neurite outgrowth (Kim et al., 2018). Therefore, although general brain morphology is not remarkably altered in *Kcnip3*^-/-^ rats, structural and morphological changes occurring in the central nervous system of knockout rats might affect central nociceptive and emotional processing, and detailed studies are needed to elucidate this issue.

### The Potential Role of KChIP3 in Dopamine Neurotransmission

RNA-Seq analysis revealed genes involved in synaptic transmission, including *Cplx3, Pcdhb3, Pcdhb22* and *Pcdhb20*. In particular, dopaminergic neurotransmission might be affected by *Kcnip3* gene deletion. *Nr4a2* and *Ddc* are associated with dopamine biosynthetic processes. *Itgad* and *Sncaip* are associated with dopamine metabolic processes. *Rgs9* is related to the modulation of dopamine receptor signaling. Notably, downregulation of *Ddc* might decrease the biosynthesis of dopamine and serotonin, which play key roles in motivation, reward and emotional processing. Therefore, decreased dopaminergic and serotonergic neurotransmission might contribute to the enhanced pain-induced aversion, anxiety- and depression-like behaviors in *Kcnip3*^-/-^ rats under both basal and inflammatory pain conditions.

Previous studies demonstrated that the KChIP3 protein was localized in the cell bodies and processes of dopaminergic neurons in the midbrain (Duncan et al., 2009). Moreover, regulation of KChIP3, particularly in mesocortical dopamine neurons, may be part of the action of antipsychotic drugs, such as haloperidol. In addition, L-DOPA-induced dyskinesia was decreased in daDREAM mice, while genetic deletion of *Kcnip3* potentiated the intensity of dyskinesia. The KChIP3 protein plays a protective role in L-DOPA-induced dyskinesia in mice (Ruiz-DeDiego et al., 2015). In addition to its transcriptional regulatory function, KChIP3 participates in the modulation of ion channels. A-type potassium channel complex formed by Kv4.3 and KChIP3 plays a key role in the pacemaker control of firing rates of dopaminergic substantia nigra neurons, which correlates with dopamine release (Liss et al., 2001). Taken together, dopaminergic neurotransmission might be affected by *Kcnip3* gene deletion, which might cause changes in emotional processing in the brain.

### A Comparison Between the Previous Study and the Current Study

Previously, Mellstrom et al. (2014) performed cDNA microarray analysis of gene expression profiles in the hippocampus of daDREAM mice. They found that gene expression of *Ctgf* and *Tshz2* was decreased while gene expression of *Col3a1, Pcdhb3* and *Ddc* was increased in the transgenic mice. Consistent with these findings, our RNA-Seq analysis revealed the upregulation of *Ctgf* and *Tshz2* gene expression and downregulation of *Col3a1, Pcdhb3* and *Ddc* gene expression in *Kcnip3*^-/-^ rats. In addition, changes in the expression profiles of *Nr4a, Egr, Rgs, Rps, Adamts, Slc* and *Rbm* gene family members were detected in both studies (Supplementary Table S4).

## Conclusion

The behavioral tests in *Kcnip3*^-/-^ rats provide the evidence for the involvement of KChIP3 in negative emotions and possible role in central nociceptive processing. RNA-seq analysis in the forebrain cortex revealed novel potential target genes of KChIP3, which are associated with neural development, synaptic transmission, and particularly, dopaminergic neurotransmission. Further studies are needed to elucidate the molecular mechanism of the emotional changes in *Kcnip3*^-/-^ rats and the possible contribution of these target genes.

## Author Contributions

Y-PG and YZ designed the experiments. Y-PG performed the behavioral test. Y-RZ and T-TL performed the biochemical studies. Y-PG, Y-RZ, YW, and YZ analyzed the data. Y-PG, Y-RZ, YW, and YZ wrote the manuscript.

## Conflict of Interest Statement

The authors declare that the research was conducted in the absence of any commercial or financial relationships that could be construed as a potential conflict of interest. The handling Editor and reviewer JN declared their involvement as co-editors in the Research Topic, and confirm the absence of any other collaboration.

## Footnotes

1 www.ehbio.com/ImageGP

## Supplementary Material

FIGURE S1 PCR analysis of CRISPR/Cas9-mediated gene deletion of *Kcnip3* **(A)** and possible off-target effects **(B)**. Arrows indicate the size of PCR products. The sequence of target primers are: forward, ATGAACAAGGCAGGGCTCACT; reverse ATGTTCAAAATAGCTCTGCGGGT; The sequence of off-target primers are: forward, TGGGTGAGCCACCAGGATGAT; reverse, TCTCCCACTGACTGGATGTGG. Touch down PCR procedure was used as follows: incubation at 95°C for 5 min, 20 cycles of 98°C for 30 s, 65°C for 30 s, 72°C for 45 s followed by 20 cycles of 98°C for 30 s, 55°C for 30 s, 72°C for 45 s, and lastly 72°C for 5 min. WT, wild-type. KO, knockout.

FIGURE S2 Western blot analysis ruled out the recognition of KChIP3 by anti-pan KChIP antibody. **(A)** Western blot analysis in N2a cells transfected with GFP or GFP-KChIP3 plasmid (as described in our previous studies performed by Na-Xi Tian et al., 2018). Expression of KChIP3 protein can be detected by anti-KChIP3 antibody, but not anti-pan KChIP antibody, in the GFP-KChIP3 transfected group. 1 μg GFP or GFP-KChIP3 plasmid was transfected with jetPRIME reagent (Polyplus, NY, United States) into N2a cells and the cells were harvested 24 h later. **(B)** Expression of KChIP3 in the spinal cord of wild-type rats could be detected by anti-KChIP3 antibody, but not anti-pan KChIP antibody. *Kcnip3* gene deletion leads to absence of KChIP3 protein in the knockout group.

FIGURE S3 qPCR analysis of *Kcnip1, Kcnip2* and *Kcnip4* **(A)**, *Pdyn* and *Bdnf* **(B)** expression in the forebrain cortex of wild-type (WT) and *Kcnip3*^-/-^ rats. *n* = 6 for both groups **(A)**. n = 5 for both groups **(B)**.

TABLE S1 Upregulated genes in the forebrain cortex of *Kcnip3*^-/-^ rat compared to that of wild-type rats.

TABLE S2 Downregulated genes in the forebrain cortex of *Kcnip3*^-/-^ rat compared to that of wild-type rats.

TABLE S3 Sequence of primers used for real-time quantitative PCR.

TABLE S4 A comparison of the differentially expressed genes in *Kcnip3*^-/-^ rats and that in daDREAM mice.
