# Supplementary figures and images for: Global Gene Knockout of Kcnip3 Enhances Pain Sensitivity and Exacerbates Negative Emotions in Rats
Source: Front Mol Neurosci. 2019 Jan 25;12:5. doi: 10.3389/fnmol.2019.00005 (PMC6355686; doi:10.3389/fnmol.2019.00005)

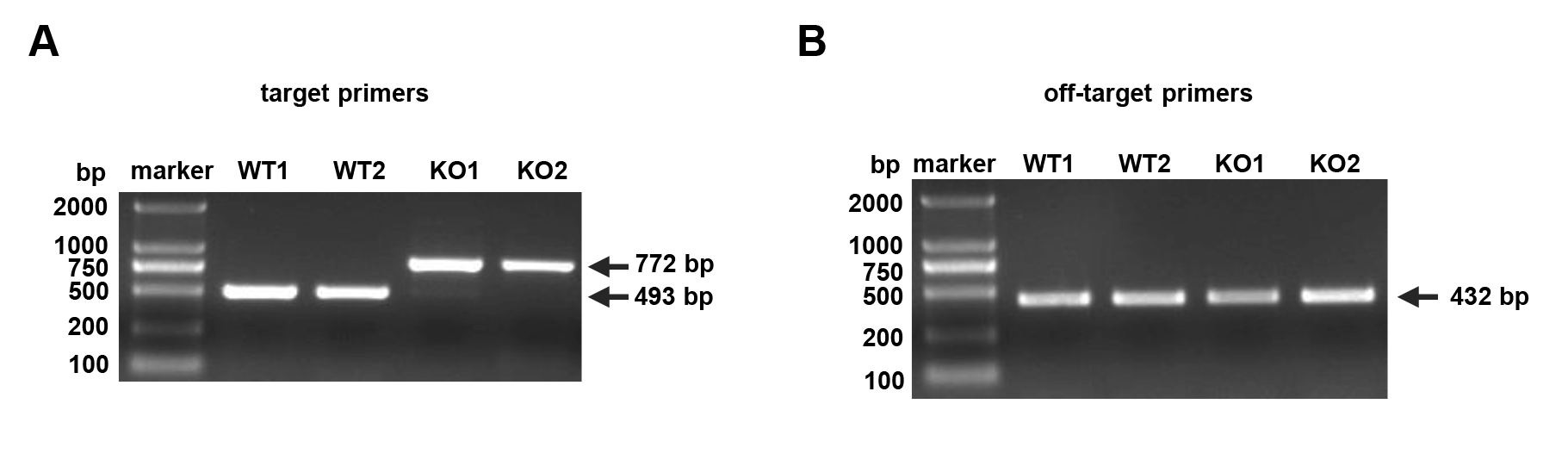

Supplement: FIGURE S1 — PCR analysis of CRISPR/Cas9-mediated gene deletion of Kcnip3 (A) and possible off-target effects (B). Arrows indicate the size of PCR products. The sequence of target primers are: forward, ATGAACAAGGCAGGGCTCACT; reverse ATGTTCAAAATAGCTCTGCGGGT; The sequence of off-target primers are: forward, TGGGTGAGCCACCAGGATGAT; reverse, TCTCCCACTGACTGGATGTGG. Touch down PCR procedure was used as follows: incubation at 95°C for 5 min, 20 cycles of 98°C for 30 s, 65°C for 30 s, 72°C for 45 s followed by 20 cycles of 98°C for 30 s, 55°C for 30 s, 72°C for 45 s, and lastly 72°C for 5 min. WT, wild-type. KO, knockout. [file Image_1.TIF]

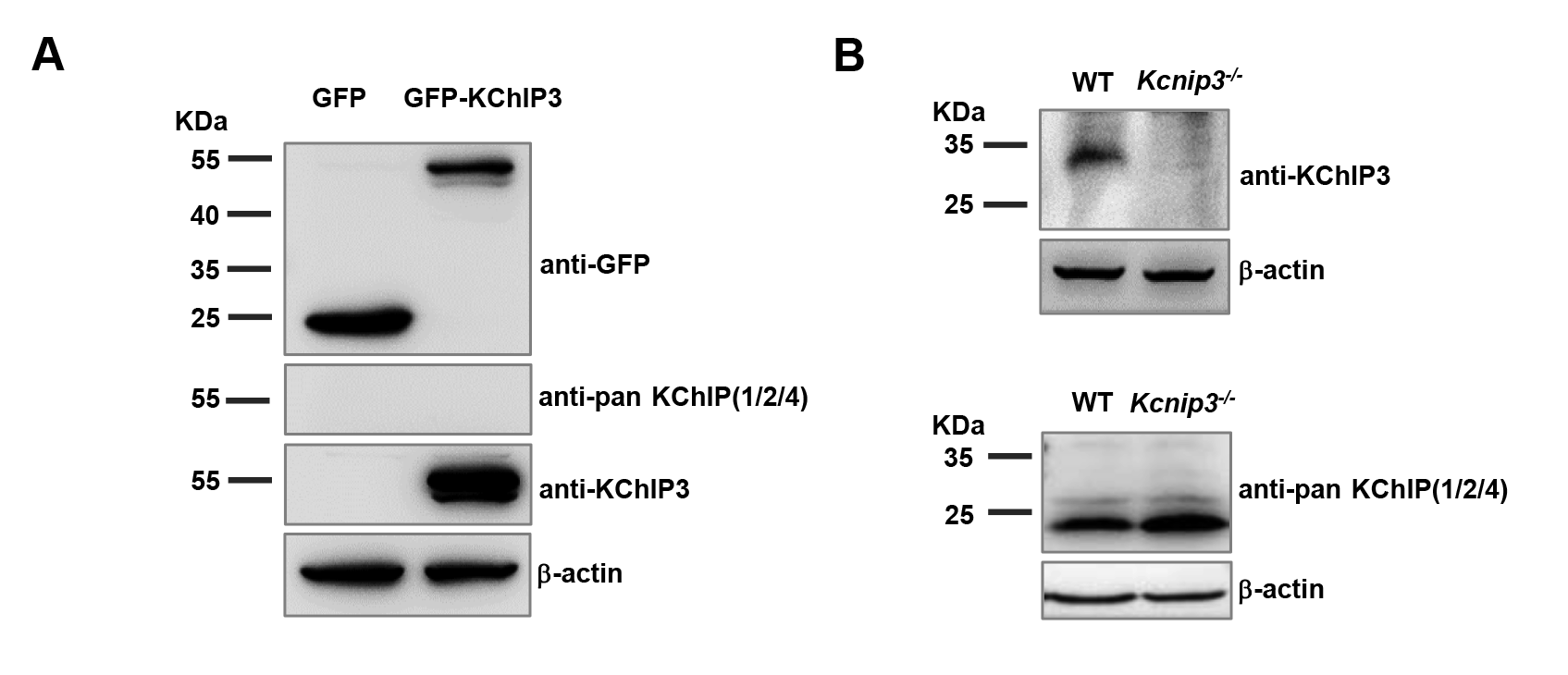

Supplement: FIGURE S2 — Western blot analysis ruled out the recognition of KChIP3 by anti-pan KChIP antibody. (A) Western blot analysis in N2a cells transfected with GFP or GFP-KChIP3 plasmid (as described in our previous studies performed by Na-Xi Tian et al., 2018). Expression of KChIP3 protein can be detected by anti-KChIP3 antibody, but not anti-pan KChIP antibody, in the GFP-KChIP3 transfected group. 1 μg GFP or GFP-KChIP3 plasmid was transfected with jetPRIME reagent (Polyplus, NY, United States) into N2a cells and the cells were harvested 24 h later. (B) Expression of KChIP3 in the spinal cord of wild-type rats could be detected by anti-KChIP3 antibody, but not anti-pan KChIP antibody. Kcnip3 gene deletion leads to absence of KChIP3 protein in the knockout group. [file Image_2.TIF]

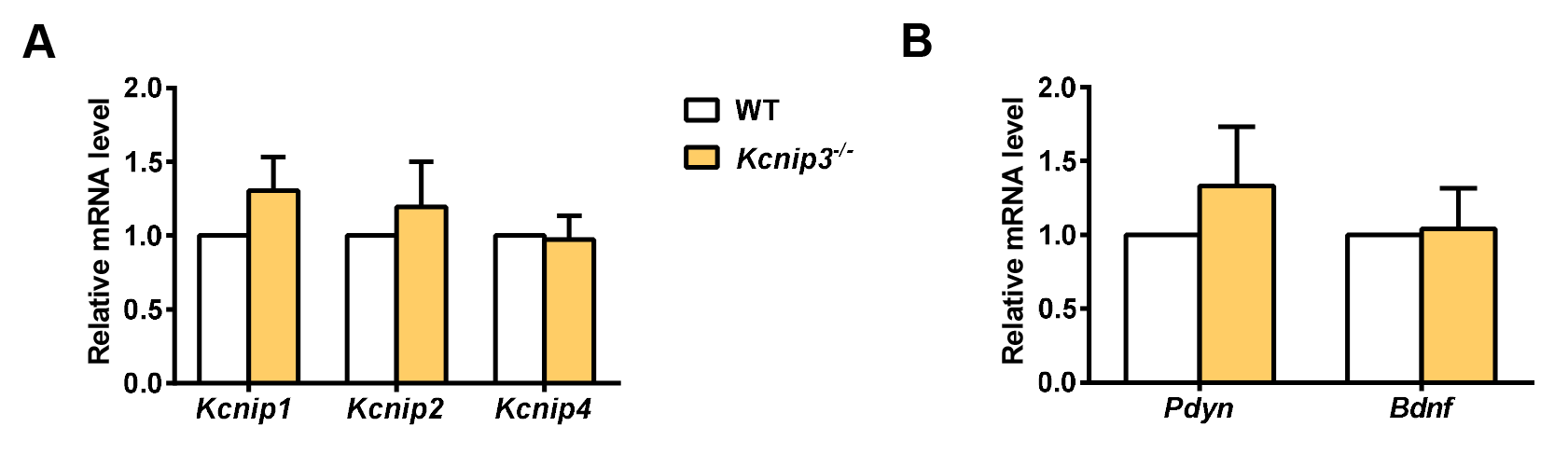

Supplement: FIGURE S3 — qPCR analysis of Kcnip1, Kcnip2 and Kcnip4 (A), Pdyn and Bdnf (B) expression in the forebrain cortex of wild-type (WT) and Kcnip3-/- rats. n = 6 for both groups (A). n = 5 for both groups (B). [file Image_3.TIF]

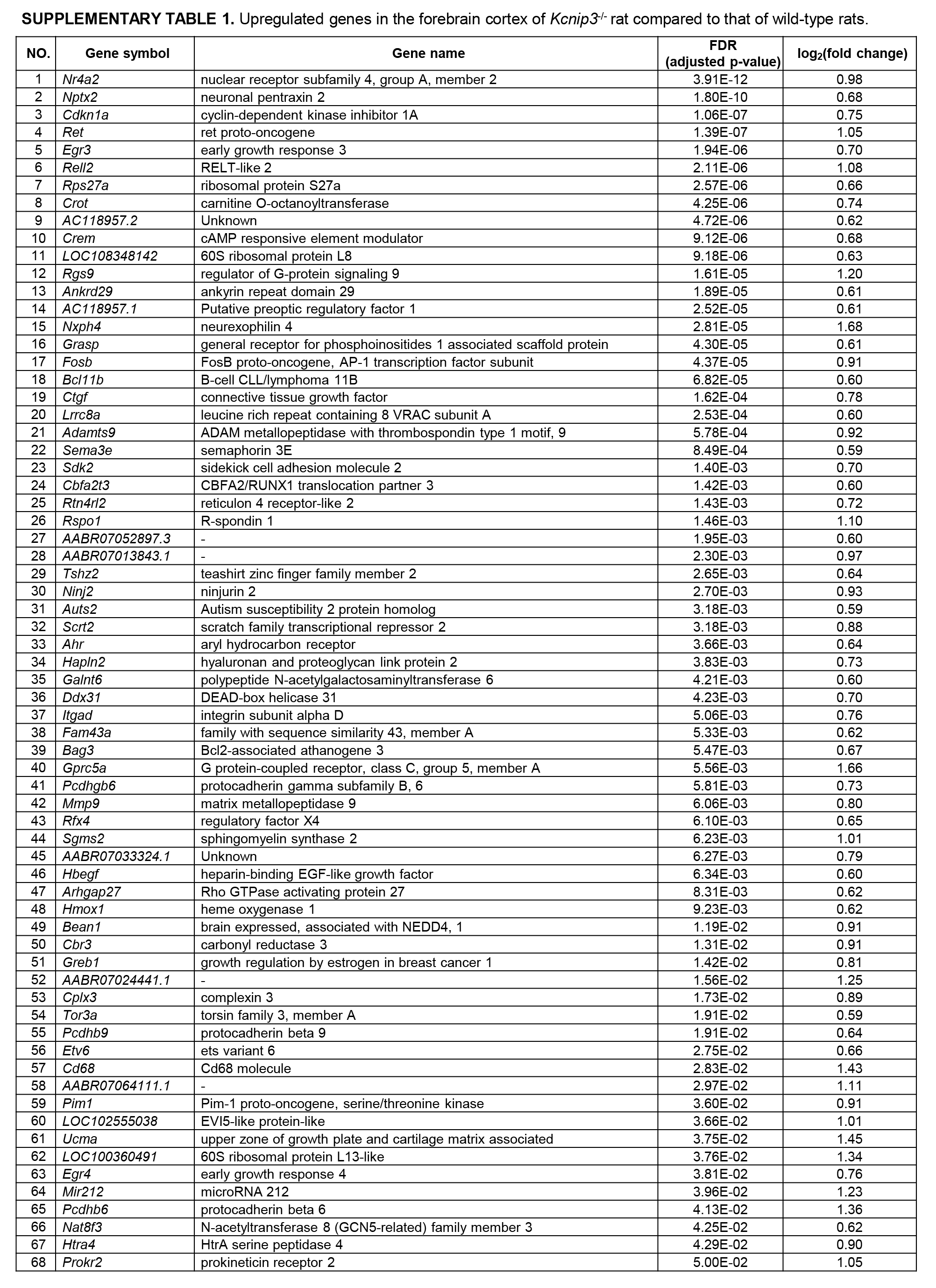

Supplement: TABLE S1 — Upregulated genes in the forebrain cortex of Kcnip3-/- rat compared to that of wild-type rats. [file Image_4.TIF]

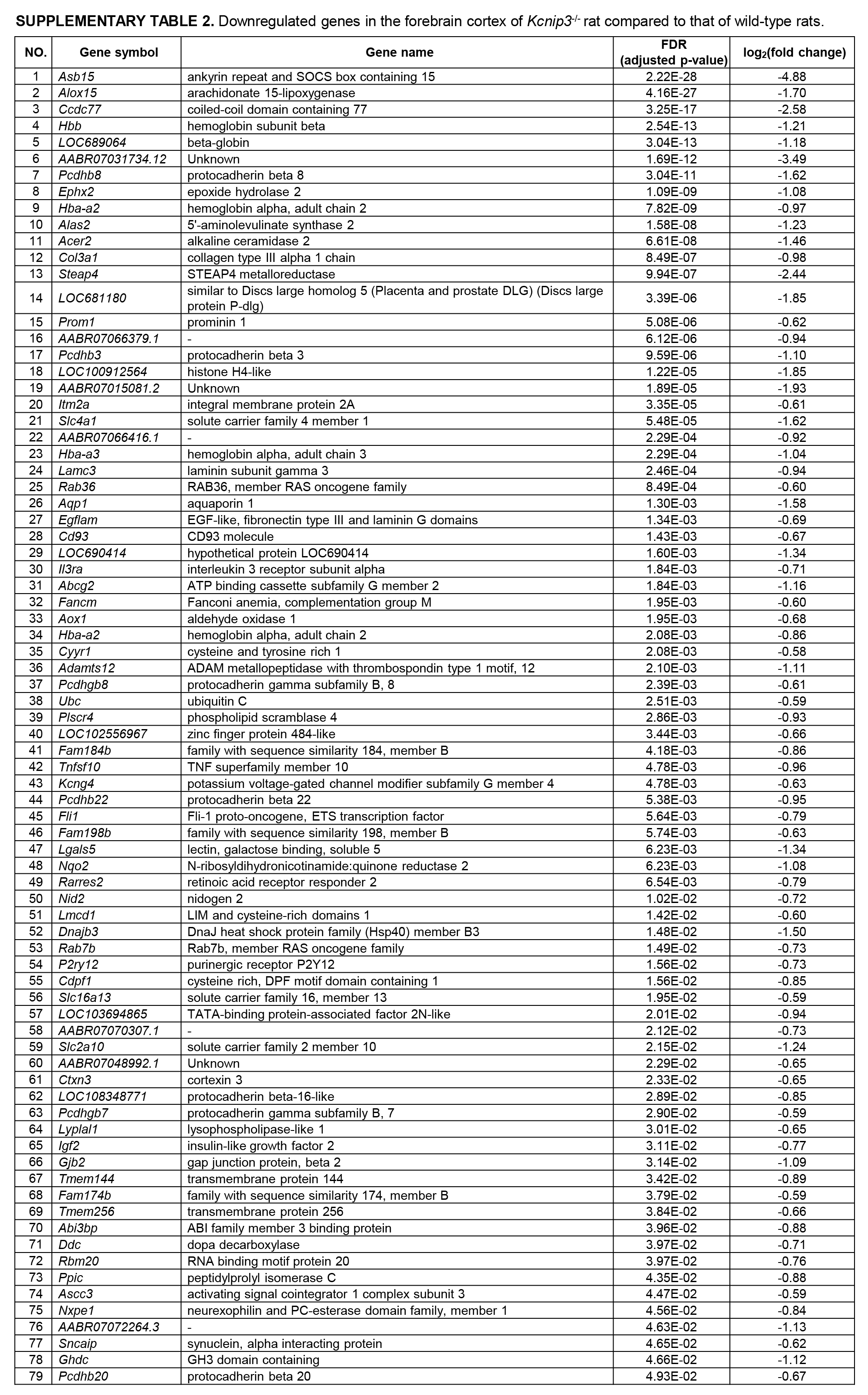

Supplement: TABLE S2 — Downregulated genes in the forebrain cortex of Kcnip3-/- rat compared to that of wild-type rats. [file Image_5.TIF]

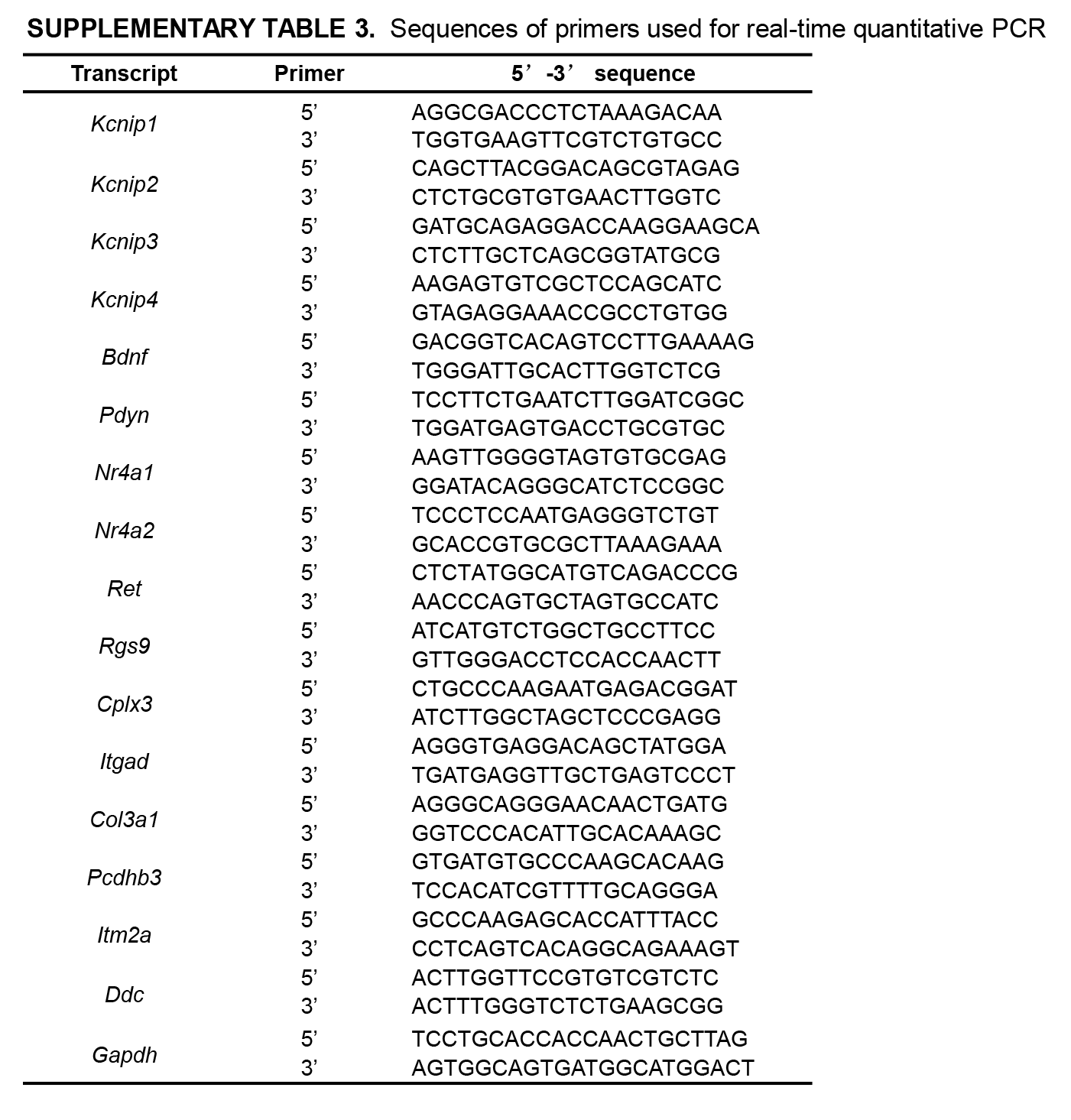

Supplement: TABLE S3 — Sequence of primers used for real-time quantitative PCR. [file Image_6.TIF]

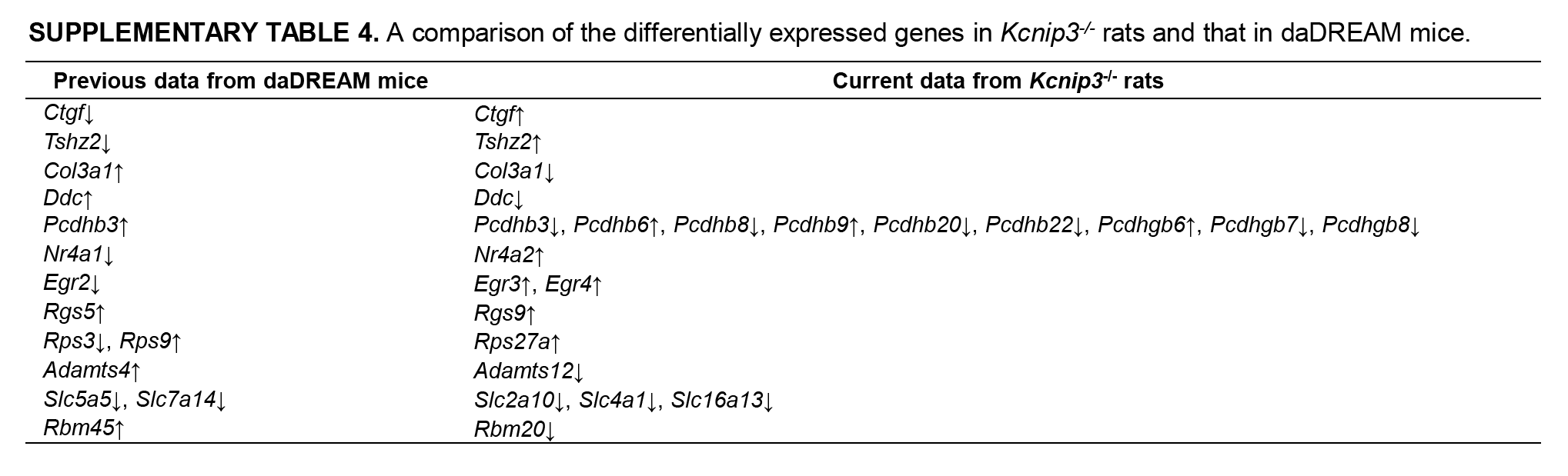

Supplement: TABLE S4 — A comparison of the differentially expressed genes in Kcnip3-/- rats and that in daDREAM mice. [file Image_7.TIF]
